# Point-of-care Ultrasound in Pregnancy: Think Congenital Zika Virus

**DOI:** 10.5811/cpcem.2016.11.32942

**Published:** 2017-01-17

**Authors:** Siri Shastry, Kristi L. Koenig

**Affiliations:** University of California, Irvine, Department of Emergency Medicine, Irvine, California

Starting in 2015, microcephaly associated with Zika virus emerged as a public health emergency of international concern. Initial cases in the United States were travel-associated; however, there are increasing reports of local transmission in pockets of the country, and therefore public concerns may escalate.^1^ Emergency physicians commonly perform point-of-care ultrasound (POCUS) on pregnant patients, a population of special concern. This paper describes ultrasound findings typical of Zika-related congenital malformations that may be incidental findings or detected when examining exposed or concerned patients during routine POCUS testing. These concerns should alert emergency physicians to assess for Zika-virus risk factors and provide urgent referral for confirmatory studies and counseling if indicated.

Fetal microcephaly and other congenital malformations are devastating consequences of Zika virus infection.^2^ As the number of cases of Zika virus within the United States increases from either travel or local transmission, emergency physicians (EP) may encounter an increased population of pregnant females with a history of exposure to the virus either via mosquito bites, sexual transmission or possibly even transfusion-related. Pregnant patients may present to the emergency department (ED) either with perceived or real exposures to Zika virus infection. Point-of-care ultrasound (POCUS) performed either on these concerned patients or on any pregnant patient for routine indications may reveal incidental findings consistent with Zika-associated congenital abnormalities. As up to 80% of patients infected with Zika virus are asymptomatic, pregnant patients may be unaware they have contracted the virus.^3^ Further, any pregnant female with a possible exposure to Zika virus may present with questions regarding the nature of possible complications as well as regarding follow-up care. Therefore, for purposes of counseling and referral, it is important that EPs be knowledgeable about ultrasound findings associated with congenital Zika virus infection as well as indications for urgent referral for confirmatory testing.

Ultrasound findings in fetuses with congenital Zika virus infection include microcephaly, intracranial calcifications, ventriculomegaly and arthrogyposis, as well as abnormalities of the corpus callosum, cerebrum/cerebellum and eyes (Images 1 and 2).^4^,^5^ Fetal microcephaly is defined as a head circumference measurement either less than two standard deviations below the average head circumference or below the third percentile for sex and gestational age.^6^ Microcephaly can be detectable as early as 18–20 weeks gestational age. Diagnostic utility of US increases as gestational age increases. Even in the absence of microcephaly, the presence of intracranial calcifications prior to 22 weeks gestational age may predict its future development.^4^ While presence of suspicious findings should prompt urgent referral, regardless of bedside US appearance, all women concerned about Zika virus infection should be referred to an obstetrician for further testing and formal US evaluation. Timing of exposure/presentation as well as serologic testing results will determine whether routine obstetric US or serial US are indicated.^5^

While the detection of specific point-of-care fetal ultrasound findings associated with Zika virus disease is beyond the scope of knowledge expected for EPs, any unusual appearance should prompt an assessment of the patient’s risk factors for the infection. This is particularly critical as the vast majority of patients will be asymptomatic. Through knowledge of US findings indicative of Zika-virus related congenital malformations, EPs will be empowered to facilitate timely referrals for concerned or possibly exposed pregnant patients who may otherwise only present to the ED for routine POCUS testing.

## Figures and Tables

**Image 1:**
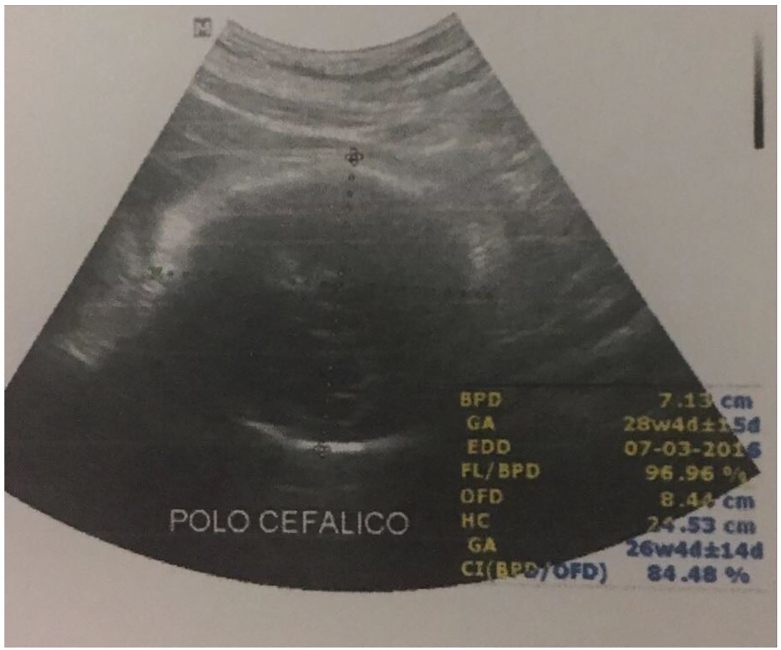
Obstetric ultrasound image depicting fetal microcephaly in a mother with a history of Zika virus infection. The fetus is at approximately 34 weeks gestational age with a head circumference of 24.53 cm. The 50th percentile head circumference at 33 weeks and 6 days is around 30 cm (Image courtesy of Dr. Edson Silva, Rio de Janeiro, Brazil).^7^

**Image 2:**
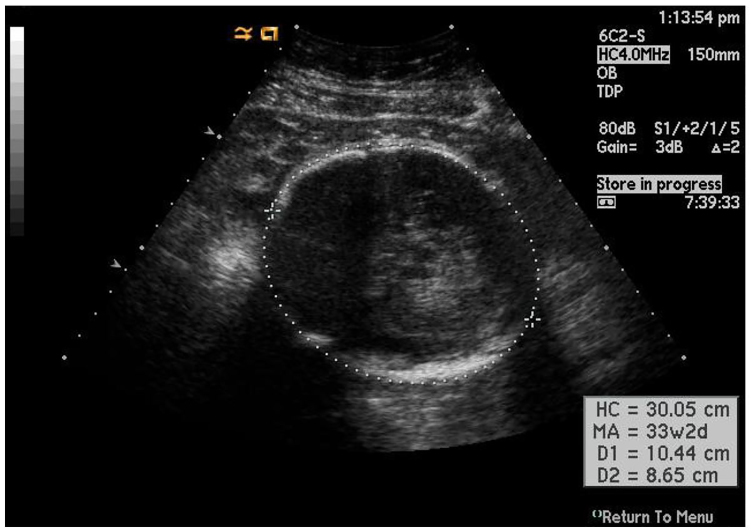
Obstetric ultrasound image depicting normal fetal anatomy at a gestational age of approximately 33 weeks and 2 days, with head circumference of 30.05 cm (Image courtesy of http://www.learnobultrasound.com/datinggrowth.htm).
